# Should the Erector Spinae Plane Block Be Applied in the Pain Management of Percutaneous Nephrolithotomy?

**DOI:** 10.7759/cureus.22554

**Published:** 2022-02-24

**Authors:** Seyma Unal, Semih Baskan, Betul Guven Aytac, Ismaıl Aytac, Melih Balci

**Affiliations:** 1 Anesthesiology, Emet State Hospital, Kütahya, TUR; 2 Anesthesiology and Critical Care, Yıldırım Beyazıt University, Ankara, TUR; 3 Anesthesiology and Reanimation, Ankara City Hospital, Ankara, TUR; 4 Urology, Ankara City Hospital, Ankara, TUR

**Keywords:** perioperative pain management, riker sedation-agitation scale, peak expiratory flow rate (pefr), percutaneous nephrolithotomy (pcnl), erector spinae plane block (espb)

## Abstract

Introduction

This prospective, randomized controlled study aimed to investigate the efficacy and respiratory effects of postoperative pain management with an erector spinae plane block in patients undergoing percutaneous nephrolithotomy.

Methods

Sixty American Society of Anesthesiologists (ASA) I-II patients aged 18-65 years, scheduled to undergo percutaneous nephrolithotomy, were randomized either to the erector spinae plane block (ESPB) or control group. Fifteen mL 0.5% bupivacaine at the T11 level was administered preoperatively using the in-plane technique in the ESPB group. In both groups, 1 gr of intravenous paracetamol was administered intraoperatively. Postoperative pain and agitation were evaluated using the visual analog scale (VAS), dynamic VAS at zero, six, and 24 hours, and the Riker sedation-agitation scale at the 0th hour after surgery. Peak expiratory flow rate (PEFR) and oxygen saturation (SpO_2_) were measured in preoperative examination and at the 0th, 6th, and 24th hours postoperatively. The time and number of the analgesic requirement, mobilization, and discharge time were also recorded.

Results

A significantly lower VAS and dynamic VAS were observed at the 0th, 6th, and 24th hours in the ESPB group (p<0.05 for each timepoint). The postoperative/preoperative PEFR ratio was lower and there were more agitated patients in the control group (p<0.05).

Conclusion

An erector spinae plane block may have additional clinical advantages while providing effective analgesia in patients who underwent percutaneous nephrolithotomy compared to intravenous analgesia.

## Introduction

Percutaneous nephrolithotomy (PCNL) is currently the most frequently preferred minimally invasive surgical procedure in the treatment of kidney stones. It is accepted as the first-line treatment for many kidney stones >2 cm, staghorn calculi, or when other methods of management fail [1].

Although PCNL is performed as a minimally invasive procedure, it causes severe postoperative pain due to dilatation of the renal capsule and parenchymal canal and peritubal distension of the nephrostomy tube [2]. Effective treatment of postoperative pain allows early mobilization of the patient, shortens the recovery and discharge time, prevents the development of chronic pain, and increases satisfaction and long-term quality of life [3].

Pain related to PCNL may cause nausea and vomiting, and aggressive management with opioids alone can result in respiratory depression [4]. Tramadol is a weak opioid used for postoperative pain relief without causing the respiratory depression seen with other opioids. It has common side effects, such as nausea and vomiting, and may be insufficient in postoperative analgesia [5]. Poor postoperative pain management increases the risk of postoperative pulmonary complications (PPC). In patients undergoing percutaneous nephrolithotomy, the decrease in inspiratory and vital capacity due to the close proximity of the operation to the diaphragm increases the risk of atelectasis [6]. In addition, unsuccessful pain management can cause postoperative delirium and agitation [7].

An erector spinae plane block (ESPB) is a peri-paravertebral regional anesthesia technique applied for the first time in the treatment of thoracic neuropathic pain [8]. ESPB, which is an easily applicable block with a low complication rate, has been shown to be effective in postoperative pain management of PCNL in the literature [9-14]. To our knowledge, this is the first study in which pain was evaluated with the dynamic visual analog scale (DVAS) in patients who underwent PCNL under ESPB, and it is also the first study to associate postoperative pain with peak expiratory flow rate (PEFR).

We aimed to show the effects of ESPB on postoperative pain management, serial PEFR, postoperative agitation score (using the Riker sedation-agitation scale), mobilization time, and length of hospital stay compared to the routine intravenous analgesia protocol in patients undergoing PCNL.

## Materials and methods

This single-center, prospective, randomized observer-blind study was conducted in Health Sciences University Ankara City Hospital between 01/03/20 and 01/08/20, after receiving approval from Ankara City Hospital's ethics committee (dated 13/02/20 and numbered E1-20-315) and registered on ClinicalTrials.gov (ID: NCT04474873).

Sixty volunteer patients of both genders, aged 18-65, in the American Society of Anesthesiologists (ASA) physical status classification I-II risk groups who were scheduled to undergo PCNL were included in the study. Three patients were excluded because of conversion to open surgery.

The exclusion criteria were ASA > II, patients with comorbidities (cardiac, respiratory, hepatic, renal, neurologic, and psychiatric), pregnancy, morbid obesity, and patients’ refusal to participate in the study.

After creating two sets of 30 unique numbers from 1 to 60 for each group using an internet-based program (www.randomize.org), the patients were randomly allocated to the control or ESPB group.

The general anesthesia protocol was the same for both groups. Anesthesia induction was performed with 1.5-2 mg/kg propofol, 0.5 to 1 mcg/kg fentanyl and 0.6 mg/kg rocuronium, and patients were intubated. General anesthesia was maintained with the end-tidal sevoflurane concentration of 2% and 0.2-0.5 mcg/kg/min remifentanil. During the operation, mechanical ventilation was applied with a tidal volume of 6-8 ml/kg, a respiratory rate of 12 breaths/min, an inspiratory-expiratory ratio of 1/2, and an oxygen flow rate of 2.0 L/min. Sugammadex 2mg/kg was used for reversal.

**Figure 1:**
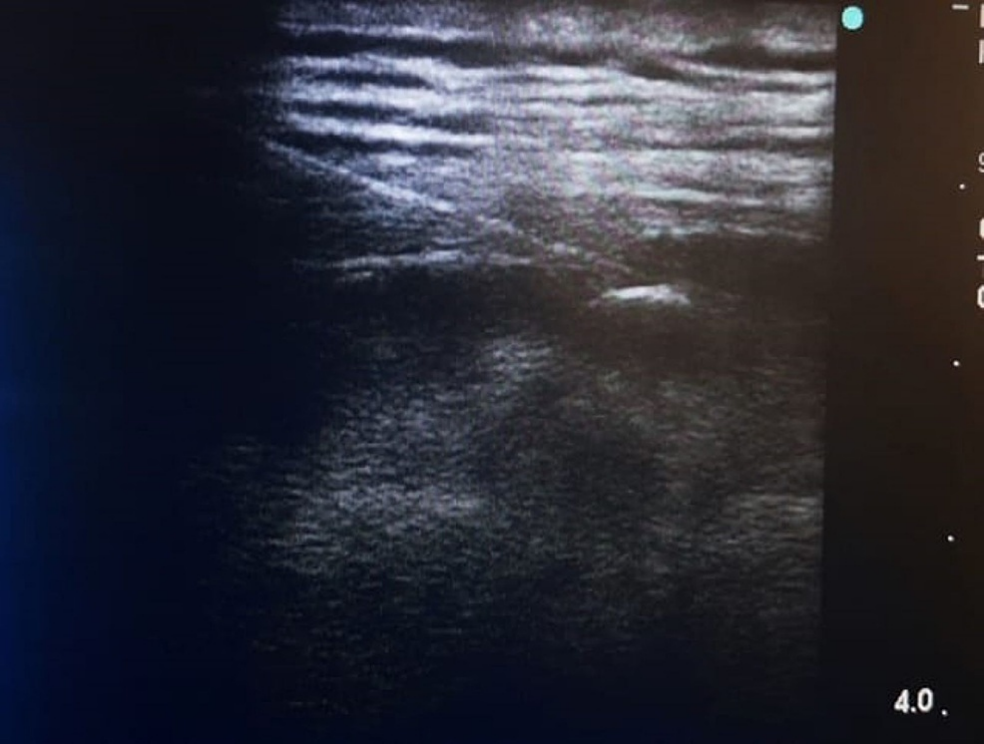
USG image of the erector spinae plane block application USG: ultrasonography

No preemptive analgesic was used in the control group. In the ESPB group, the block was performed with ultrasonography (USG; Toshiba Diagnostic Ultrasound System, GM-55402A00E, Japan, 8 mHz linear probe) before general anesthesia induction. The patients were placed in the prone position and after skin cleaning, ESPB was performed at the T11 level using the in-plane technique. Before the procedure, 3 mL of 2% lidocaine was applied locally to the patient's skin. A 21G 100 mm insulated needle 15 (Vygon Echoplex, France) was inserted in the cranial-caudal direction until it made contact with the T11 transverse protrusion in the in-plane approach. Hydrodissection was applied with a 15 ml saline solution. Then, a total of 15 mL 0.5% bupivacaine was injected as a local anesthetic. The location of the needle tip was confirmed by removing the erector spinae muscle from the bone shadow of the transverse process and observing the distribution of local anesthetic in both cranial and caudal directions (Figure 1).

**Figure 2:**
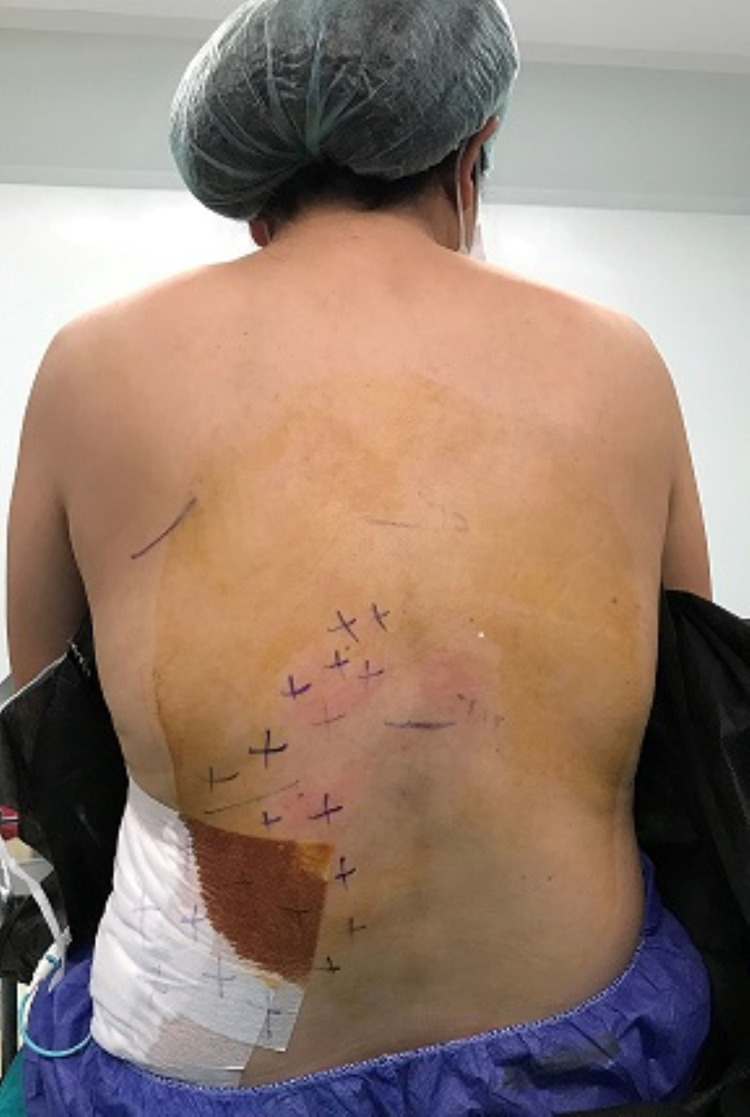
Dermatomal distribution of the sensory block evaluated by the pinprick test

The analgesic effect was evaluated by the pinprick test 20 minutes after the block intervention, including the T10, T11, and T12 nerve distribution segments (Figure 2). A successful ESPB must contain all three segments; otherwise, the block was considered unsuccessful and planned to remove from the study. All patients in the ESPB group pinprick test were considered as a successful block.

The use of the peak flow meter (ExpiRite Peak Flow Meter®, BSSMed, Istanbul, Turkey) was explained to all patients during the preoperative interview. The patients were asked to breathe as deeply as they could, and then to breathe into the flowmeter as effectively and rapidly as they could. It was explained to the patients that they should grasp the peak flowmeter with their lips in a sitting position in the bed and blow it in one breath and that they should not put their tongue on the end of the device during blowing. PEFR is the maximum rate of flow in forced expiration starting from full inspiration (15). Measurements were made three times for each visit and the highest value was recorded. PEFR values were obtained at the preoperative baseline and postoperative 0th, 6th, and 24th hours.

In both groups, 1 gr of intravenous paracetamol was administered intraoperatively during skin closure and at the 8th and 16th hours postoperatively. Postoperative pain and agitation were evaluated using VAS, dynamic VAS at zero, six, and 24 hours and the Riker sedation-agitation scale at 0th hours after surgery. PEFR and SpO2 were measured in the preoperative examination and at the 0th, 6th, and 24th hours postoperatively. In the postoperative period, intravenous tramadol (100 mg) was administered as an additional analgesic when VAS ≥ 4. The time and number of the analgesic requirement and administrations, mobilization and oral intake time, and length of hospital stay were recorded. Duration of surgery, diameter, number, and location of the renal stones were also recorded.

In all patients, the time from stopping sevoflurane inhalation to awakening was called the awakening time and was recorded. The Riker sedation-agitation scale was used to determine the anxiety level of the patients in the recovery room. Postoperative pain was evaluated using VAS and dynamic VAS (pain with deep breathing and cough-DVAS) at zero, six, and 24 hours after surgery. For VAS and DVAS, two end descriptions were written on both ends of a 100 mm line, and patients were asked to indicate where their condition was appropriate by drawing a line or by putting a dot or mark on this line. Pain due to urinary catheter application was also evaluated. The evaluations were done by a blinded observer independent of the study.

Statistical analysis

Data analysis was performed using the IBM SPSS 25.0 (Armonk, NY: IBM Corp.) statistical package program. The chi-square test was used to compare qualitative data as well as descriptive statistical methods (frequency, percentage, mean, standard deviation, median, and min-max. IQR) while evaluating the study data. The compliance of the data to normal distribution was evaluated by the Kolmogorov-Smirnow and Shapiro-Wilk tests.

In the research, in the evaluation of the quantitative data with normal distribution, the independent samples t-test (t-test in independent groups) and repeated measures analysis of variance (ANOVA) were used for the comparison of repeated measurements. The post-hoc Tukey honest significant difference (HSD) test was used to find the source of the difference in cases where there was a difference in multiple comparisons. The Mann-Whitney U test was used to evaluate data that did not show normal distribution. Relationships between variables were evaluated using the Pearson correlation test. Statistical significance level was accepted as α = 0.05. Power analysis was made with the G * Power 3.1.9.4 statistical package program. As n1 = 28, n2 = 29, α = 0.05, Effect Size (d) = 0.87; power = 90%.

## Results

There were no differences in patients' characteristics between groups (Table 1).

**Table 1:** Comparison of the patient characteristics between the control and ESPB groups a: chi-square test, b: independent samples t-test, c: Mann-Whitney U test BMI: body mass index; ESPB: erector spinae plane block; SD: standard deviation; IQR: interquartile range

		Control (n=28)	ESPB (n=29)	P
Gender (n / %)	Female	7 (25.0%)	7 (24.1%)	1.000^ a^
	Male	21 (75.0%)	22 (75.9%)	
Age (Year) (Mean ± SD)		52.0 ± 10.5	53.0 ± 10.4	0.719 ^b^
Weight (kg) (Mean ± SD)		78.9 ± 13.4	79.3 ± 13.5	0.915 ^b^
Height (cm) (Mean ± SD)		170.5 ± 9.8	169.9 ± 6.2	0.806 ^b^
BMI (kg/m^2^) (Mean ± SD)		27.1 ± 3.8	27.4 ± 4.2	0.744 ^b^
ASA (n / %)	I	2 (7.1%)	2 (6.9%)	1.000^ a^
	II	26 (92.9%)	27 (93.1%)	
Operation time(min) (Median / IQR)		155.0(115.5-174.8)	140.0(115.5-170.0)	0.363^c^
Awakening time (min) (Median / IQR)		8.0 (5.0 - 10.8)	9.0 (5.0 - 10.0)	0.981^c^
Renal stone diameter(cm) (Median / IQR)		2.1 (1.5 - 3.0)	2.5 (2.0 - 3.2)	0.215^c^
Number of the Stones (Median / IQR)		2.0 (1.0 - 4.0)	2.0 (1.0 - 4.0)	0.961^c^

VAS and DVAS values were lower in the ESPB group at the 0th, 6th, 24th hours (p<0.05) but pain related to the urinary catheter was similar between groups (Tables 2-3).

**Table 2:** Comparison of the patients’ assessments of pain between groups according to the visual analog scale score (VAS) *: Independent samples t-test ESPB: erector spinae plane block; VAS: visual analog scale; SD: standard deviation

VAS	Control (n=28)	ESPB (n=29)	P*
Recovery unit (Mean ± SD)	6.7 ± 2.1	3.0 ± 2.2	<0.0001
6^th^ hour (Mean ± SD)	4.6 ± 2.2	2.8 ± 1.9	0.002
24^th^ Hour (Mean ± SD)	2.9 ± 2.2	1.3 ± 1.4	0.001
VAS for Urinary catheter pain (Mean ± SD)	2.4 ± 4.0	3.5 ± 3.8	0.314

**Table 3:** Comparison of the patients’ assessments of pain between groups according to the dynamic visual analog scale (DVAS) score *: independent samples t-test; ESPB: erector spinae plane block; SD: standard deviation; DVAS: dynamic visual analog scale

DVAS	Control (n=28)	ESPB (n=29)	P*
Recovery unit (Mean ± SD)	7.6 ± 2,0	3.8 ± 2.3	0.0001
6^th^ hour (Mean ± SD)	5.4 ± 2.2	3.9 ± 2.4	0.020
24^th^ hour (Mean ± SD)	4.2 ± 2.7	2.0 ± 2.3	0.002

The number of agitated patients in the recovery room was higher in the control group (11 (39.3%); 4 (13.8%), p<0.005) (Table 4).

**Table 4:** Comparison of the groups according to the Riker agitation sedation scale *: chi-square test

	Control (n=28)	ESPB (n=29)	P*
RIKER≤ 4	17 (60.7%)	25 (86.2%)	0.027
RIKER >4	11 (39.3%)	4 (13.8%)	

The number of and time to additional analgesic requirement and administration (100 mg tramadol) were lower in the ESPB group within 24 hours (p<0.005) but oral intake time, mobilization time, and length of hospital stay were similar between groups (Table 5).

**Table 5:** Comparison of postoperative characteristics between groups *: Mann-Whitney U test; ESPB: erector spinae plane block; IQR: interquartile range

		Control (n=28)	ESPB (n=29)	P^*^
Time to Analgesic requirement(h)(Median / IQR)		0.41 (0.16 - 1.43)	1.60 (0.75 - 5.05)	0.004
Number of the analgesic requirement(Median / IQR)		2.00 (2.00 - 3.00)	2.00 (1.00 - 2.00)	0.046
Mobilization time (h)(Median / IQR)		22.0 (18.9 - 24.0)	21.,0 (17.8 - 24.5)	0.930
Oral intake time(h)(Median / IQR)		20.5 (17.4 - 23.0)	18.0 (10.3 - 21.0)	0.092
Length of hospital stay (day)(Median / IQR)		2.5 (2.0 - 4.0)	2.0 (2.0 - 4.5)	0.913

The ratio of preoperative PEFR

Postoperative PEFR in the recovery room (0th hour) was lower in the control group compared to the ESPB group (64,5%, 74,8%; respectively, p<0.005) but it was similar in the later hours (Table 6). The SpO2 values of the patients were similar in both groups at zero and six hours but were lower in the control group at 24 hours (p<0.05).

**Table 6:** Comparison of the rate of change in PEFR values and postoperative SPO2 values between groups *: Independent samples T-test; **: repeated measures analysis of variance (ANOVA) test; ^a^: p-value is derived from a comparison of 1-2 and a comparison of 1-3, ^b^: p-value is derived from a comparison of 1 and 3 1: Percentage of the ratio of the 0th-hour postoperative peak expiratory flow rate in the recovery room to the preoperative peak expiratory flow rate 2:Percentage of the ratio of the 6th-hour postoperative peak expiratory flow rate in the recovery room to the preoperative peak expiratory flow rate 3: Percentage of the ratio of the 24th-hour postoperative peak expiratory flow rate in the recovery room to the preoperative peak expiratory flow rate ESPB: erector spinae plane block; SD: standard deviation

	Control (n=28)	ESPB (n=29)	P*
PEFR0/ PEFR_preop_(%) (Mean ± SD)	64.5 ± 15.4	74.8 ± 19.0	0.029
PEFR6 / PEFR_preop_(%)(Mean ± SD)	73.5 ± 17.4	79.6 ± 15.7	0.167
PEFR24 / PEFR_preop_(%) (Mean ± SD)	79.5 ± 18.7	84.5 ± 15.8	0.286
P**	<0.0001 ^a^	0,018^b^	
SpO_2 _(Recovery Room)(Mean ± SD)	95.1 ± 3.1	96.5 ± 2.,0	0.051
SpO_2_ (Postop 6.h)(Mean ± SD)	95.9 ± 2.9	96.6 ± 2.0	0.276
SpO_2_ (Postop 24. h)(Mean ± SD)	95.8 ± 2.9	97.2 ± 1.3	0.027

## Discussion

In our study, we observed that ESPB reduces the incidence of agitation and prevents PEFR reduction compared to preoperative values in the recovery room, thanks to the positive effects of PCNL on postoperative pain management. ESPB provided low VAS and DVAS values within the first 24 hours, and the mean SPO2 values of the patients were found to be higher at the 24th hour compared to the control group.

Regional analgesia is an important element of successful postoperative pain management since they reduce the consumption of opioids, which have a high profile of side effects such as respiratory depression, nausea, vomiting, and slowing bowel movements [15]. The trend in postoperative pain management has turned from epidural analgesia to the paravertebral, truncal, and recently erector spinae plane block since it can be applied easily and has fewer complications [16-17].

ESPB was defined by Forero et al. for the treatment of neuropathic chest pain in 2016 and has become popular as a postoperative pain treatment in many surgical procedures. It is a good alternative because of its relatively easy application compared to paravertebral blocks, and it does not have complications such as pneumothorax, subarachnoid injection, urinary retention, and hypotension.

There is rapidly expanding literature on the efficacy of ESPB in postoperative pain management of the PCNL [9-14]. Apart from case reports, two randomized controlled studies [9,11], have been published on this subject. Ibrahim et al. compared the efficacy of ESPB with the control group in 50 patients who had undergone PCNL operation [9]. They showed that ESPB reduces intraoperative fentanyl and postoperative 24-hour morphine consumption and numerical rating scale (NRS) scores were significantly lower at the 2nd and 12th hours in the ESPB group. Also, Gultekin et al. compared the administration of ESPB with conventional intravenous analgesia in a randomized controlled study of 60 patients undergoing PCNL surgery [11]. They showed that VAS scores in the first 24 hours were significantly lower in the ESPB group, the duration of first analgesic use was prolonged, and total tramadol and paracetamol consumption decreased.

Our study may contribute to the relevant literature in several points. We ruled out potential confusion in the subjective evaluation of the patients by questioning the pain caused by the urinary catheter. We comprehensively investigated the pain using serial PEFR measurements and DVAS in association with its features that may be related to cough, respiration, and mobilization in addition to the subjective and one-dimensional VAS scale. Additionally, we investigated the positive outcomes of pain management with serial PEFR-SpO2 measurements, assessing recovery agitation, mobilization, oral intake, and discharge time.

After thoracic and upper abdominal surgeries, it has been shown that pain affects respiratory muscles and impairs respiratory functions. Pain can reduce vital capacity, may cause the development of atelectasis and postoperative hypoxemia [18].

PEFR is an inexpensive, easily accessible respiratory function test that reflects vital capacity. PEFR value may decrease in the early postoperative period due to pain [19]. In PNL surgery, Hosseini et al. investigated the effectiveness of peritubal ketamine infiltration and Imani et al. also investigated the analgesic efficacy of ropivacaine infiltration and its effects on PEFR values. In these studies, it was shown that PEFR values decreased significantly in the early postoperative period after PNL, but pain management with peritubal infiltration did not affect PEFR values positively [4,19].

Our study showed that ESPB provides effective pain management and improves patient outcomes by preventing agitation and negative effects on early pulmonary functions.

For all that, there are several limitations of our study. Patient-controlled analgesia methods may be preferred instead of intermittent intravenous additional tramadol analgesia. It can be assumed that this can reduce the adverse outcomes of the intermittent bolus intravenous opioids. The exclusion of patients with ASA > II and the small sample size may have caused us not to measure the effect of ESPB on the length of hospital stay and mobilization time.

## Conclusions

ESPB is an effective alternative in postoperative pain management for PCNL. It provides sufficient analgesia within the first 24 hours after surgery and may reduce adverse outcomes related to the pain such as emergence agitation and PEFR reduction.
